# Association Between Acute-Phase Occupational Therapy and Quality of Life at Hospital Discharge and 90 Days After Discharge

**DOI:** 10.3390/jcm15187229

**Published:** 2026-09-17

**Authors:** Seiya Sato, Nobuto Nakanishi, Shinichi Watanabe, Kohei Tanaka, Yasunari Morita, Keibun Liu, Daisetsu Yasumura, Nanba Tomoya, Gen Kudo, Ryo Kozu, Kenzo Ishii, Kensuke Nakamura, Taku Oshima, Hajime Katsukawa

**Affiliations:** 1 Division of Disaster and Emergency Medicine, Department of Surgery Related, Kobe University Graduate School of Medicine, Kobe 650-0017, Japan; seiya033173@gmail.com (S.S.);; 2 Department of Rehabilitation Medicine, National Hospital Organization Nagoya Medical Center, Nagoya 460-0001, Japan; 3 Department of Rehabilitation Medicine, Osaka International Medical & Science Center, Osaka 558-8558, Japan; 4 Department of Emergency and Intensive Care Medicine, National Hospital Organization Nagoya Medical Center, Nagoya 460-0001, Japan; 5 Department of Academic Research, Non-Profit Organization ICU Collaboration Network (ICON), Tokyo 113-0033, Japan; 6 Department of Rehabilitation, Naha City Hospital, Naha 902-8511, Japan; 7 Department of Rehabilitation, Kindai University Nara Hospital, Nara 630-0293, Japan; 8 Department of Rehabilitation, Tokyo Women’s Medical University, Tokyo 162-8666, Japan; gensuke.3ys@gmail.com; 9 Department of Rehabilitation Medicine, Nagasaki University Hospital, Nagasaki 852-8501, Japan; ryokozu@nagasaki-u.ac.jp; 10 Department of Physical Therapy Science, Nagasaki University Graduate School of Biomedical Sciences, Nagasaki 852-8523, Japan; 11 Surgical and Medical Intensive Care Unit, Fukuyama City Hospital, Fukuyama 721-8511, Japan; 12 Japanese Society for Early Mobilization, Tokyo 102-0073, Japan

**Keywords:** occupational therapy, intensive care unit, critical illness, post-intensive care syndrome, quality of life, rehabilitation, early mobilization, EQ-5D-5L

## Abstract

**Background/Objectives**: Early rehabilitation in critically ill patients has been associated with improved physical outcomes. However, evidence regarding the association between occupational therapy and quality of life (QOL) remains limited. This study examined the association between acute-phase occupational therapy and QOL. **Methods**: Data were obtained from the multicenter prospective EMPICS study. Patients who remained in the intensive care unit (ICU) for at least 48 h were included. QOL was assessed using the EuroQol 5-Dimensions 5-Levels (EQ-5D-5L) at hospital discharge and 90 days after discharge. Inverse probability of treatment weighting analyses examined the associations between occupational therapy and EQ-5D-5L outcomes. **Results**: A total of 178 patients were included (physical therapy group, *n* = 138; physical plus occupational therapy group, *n* = 40). Occupational therapy was independently associated with a more favorable EQ-5D-5L index score at hospital discharge (B = 0.13, 95% CI: 0.02–0.24; *p* = 0.02). Patients who initiated occupational therapy during ICU stay had significantly more favorable EQ-5D-5L index score at discharge than those who started after ICU discharge (B = 0.22, 95% CI: 0.07–0.36; *p* < 0.01). **Conclusions**: Acute-phase occupational therapy, particularly when initiated during ICU stay, was associated with better QOL at hospital discharge but not at 90 days. Further studies are needed to clarify its long-term clinical benefits.

## 1. Introduction

It has been reported that more than half of intensive care unit (ICU) survivors experience post-intensive care syndrome (PICS) or impairment in at least one functional domain within one year after ICU discharge [1]. PICS is associated with reduced quality of life (QOL). At six months after ICU discharge, 77% of patients reported moderate or severe impairment in at least one domain of the EuroQol 5-Dimensions 5-Levels (EQ-5D-5L). Specifically, moderate to severe impairment was observed in anxiety/depression (54%), usual activities (46%), pain/discomfort (45%), mobility (37%), and self-care (22%) [2]. Furthermore, reduced QOL persisted in 63% of patients one year after ICU discharge [3]. These findings indicate that ICU survivors frequently experience persistent PICS and long-term impairment in QOL.

Early rehabilitation during ICU stay has received increasing attention as a strategy to prevent or mitigate PICS [4]. Previous studies have shown that early rehabilitation shortens the duration of delirium and improves activities of daily living (ADL) at hospital discharge. Schweickert et al. demonstrated that early rehabilitation was associated with an approximately 25-point improvement in the Barthel Index at hospital discharge [5]. Another study reported that 66% of patients achieved independence on the Functional Independence Measure (FIM) by discharge following early rehabilitation [6]. These findings suggest beneficial effects on short-term functional outcomes. Moreover, early rehabilitation has also been associated with improved long-term health-related QOL. One study demonstrated significant improvements in the physical functioning (81.8 ± 22.2 vs. 60.0 ± 29.4) and role limitations due to physical health (61.4 ± 43.8 vs. 17.1 ± 34.4) of the Short Form-36 Health Survey (SF-36) at six months after discharge [7]. Taken together, early rehabilitation may improve both short- and long-term outcomes.

However, in most studies of early rehabilitation in the ICU, occupational therapy has been evaluated together with physical therapy, and evidence regarding the independent effects of occupational therapy on PICS remains limited. The EFFORT-ICU trial, a randomized controlled trial (*n* = 30), evaluated early enhanced occupational therapy in mechanically ventilated ICU patients. However, no significant differences were observed in QOL at hospital discharge, as measured by the SF-36 physical component summary (31.5 vs. 38.0) or mental component summary (47.4 vs. 51.6) [8]. A subsequent 90-day follow-up in a limited sample (11 patients in the intervention group and 9 in the control group) suggested a potential improvement in FIM motor scores; however, the small sample size limited definitive conclusions. Therefore, the independent contribution of occupational therapy to short- and long-term outcomes in acute care patients, including ICU survivors, remains unclear.

The present study aimed to examine the association between acute-phase occupational therapy intervention, provided during acute-care hospitalization, and QOL at hospital discharge and 90 days after discharge using data from a multicenter prospective observational study. We hypothesized that acute-phase occupational therapy intervention would be independently associated with a favorable EQ-5D-5L index score at hospital discharge and 90 days after discharge.

## 2. Materials and Methods

### 2.1. Study Design, Setting, and Patients

This study used data from the EMPICS study (Early Mobilization and Psychiatric Symptoms in ICU Survivors; UMIN ID: UMIN000036503; accessed 17 July 2025) [9]. The EMPICS study is a multicenter prospective observational study conducted in Japan to investigate the association between early mobilization and PICS-related outcomes in ICU patients. Patients were eligible for inclusion if they remained in the ICU for at least 48 h between June and December 2019. The exclusion criteria were age < 18 years, inability to walk independently before hospital admission, neurological complications, impaired communication due to pre-existing psychiatric disorders, and terminal illness or receipt of end-of-life care. For the present analysis, patients who died during their ICU stay were excluded. This study was approved by the Ethics Committee of the National Hospital Organization Nagoya Medical Center (Approval No. 2018093; approved on 15 May 2019) and by the institutional review boards of all participating hospitals.

### 2.2. Quality of Life Assessment

QOL was assessed using the EQ-5D-5L, which comprises five dimensions: mobility, self-care, usual activities, pain/discomfort, and anxiety/depression [10,11]. Each dimension is rated on a five-level scale ranging from “no problems” (level 1) to “extreme problems” (level 5) [12]. Responses were converted into country-specific index values (1, full health; 0, death; <0, worse than death). EQ-5D-5L index score was calculated using the Japanese value set [13]. Higher EQ-5D-5L index score indicates better QOL, whereas higher dimension levels indicate greater impairment. QOL was assessed at hospital discharge and 90 days after hospital discharge. The EQ-5D-5L was administered face-to-face by the treating therapist at hospital discharge and by telephone 90 days after hospital discharge.

### 2.3. Physical Function Assessment

Physical function was assessed using the Barthel Index, a widely used measure of functional ability in critically ill patients [14]. In addition, the Medical Research Council (MRC) sum score and handgrip strength were assessed [15,16]. These variables were evaluated at hospital discharge.

### 2.4. Psychological Assessment

Anxiety and depression were assessed using the Hospital Anxiety and Depression Scale (HADS), a validated self-report questionnaire for evaluating symptoms of anxiety and depression in critically ill patients [17]. The HADS consists of 14 items, including seven items for anxiety (HADS-A) and seven items for depression (HADS-D), with each item scored from 0 to 3. The maximum score for each subscale is 21, and symptoms of anxiety or depression were defined as a score of ≥8.

Post-traumatic stress disorder (PTSD) symptoms were assessed using the Impact of Event Scale—Revised (IES-R), a 22-item self-report questionnaire with total scores ranging from 0 to 88, in which each item is scored from 0 to 4 [18]. Clinically significant PTSD symptoms were defined as an IES-R score of ≥25. These assessments were performed at hospital discharge and 90 days after hospital discharge.

### 2.5. Follow-Up Protocol

At 90 days after hospital discharge, physicians, nurses, or physical therapists at each participating center contacted patients by telephone to confirm survival. If the patient was confirmed to be alive, the EQ-5D-5L questionnaire [19] was administered verbally during the same telephone interview. Subsequently, the HADS and IES-R questionnaires, together with response forms, were mailed to the patients. Completed questionnaires were returned by mail to the respective participating centers. Patients who did not respond despite multiple telephone contacts were considered lost to follow-up and were excluded from the analysis.

### 2.6. Statistical Analysis

Data are presented as median [interquartile range] or number (%). Continuous variables were compared using the Mann–Whitney U test, and categorical variables were compared using the chi-square test or Fisher’s exact test, as appropriate. The distribution of each continuous variable was assessed using the Shapiro–Wilk test before performing nonparametric analyses. As this was an observational study using a prospectively enrolled cohort, an a priori sample size calculation was not performed. The sample size was determined by the number of eligible participants enrolled during the predefined study period.

To examine the association between occupational therapy and health-related QOL ad hospital discharge, inverse probability of treatment weighting (IPTW) based on propensity scores was used. Inverse probability of censoring weighting (IPCW) was used to analyze the 90-day outcomes after hospital discharge. The propensity score model included age, sex, Charlson Comorbidity Index, APACHE II score, Barthel Index before ICU admission, admission type (emergency or elective), and hospital-level differences. In the IPTW-adjusted outcome analyses, age, sex, APACHE II score, occupational therapy, and variables with standard mean difference (SMD) > 0.1 were included as covariates in the weighted regression models to further adjust for potential confounding. Multiple linear regression analysis was performed for the EQ-5D-5L index score, whereas ordinal logistic regression analysis was performed for the individual EQ-5D-5L dimensions. Regression coefficients (B) or odds ratios (ORs) with corresponding 95% confidence intervals (CIs) were presented. Covariate balance before and after weighting was assessed using standardized mean differences.

A further analysis was conducted to investigate the effect of initiating occupational therapy in the ICU compared with initiation in the ward. Patients were categorized into two groups according to the timing of occupational therapy initiation: the ICU group (patients who initiated occupational therapy during ICU stay) and the Ward group (patients who initiated occupational therapy after ICU discharge). Group comparisons were performed using the same statistical methods described above. A two-sided *p*-value < 0.05 was considered statistically significant.

## 3. Results

### 3.1. Baseline Characteristics

**Figure 1:**
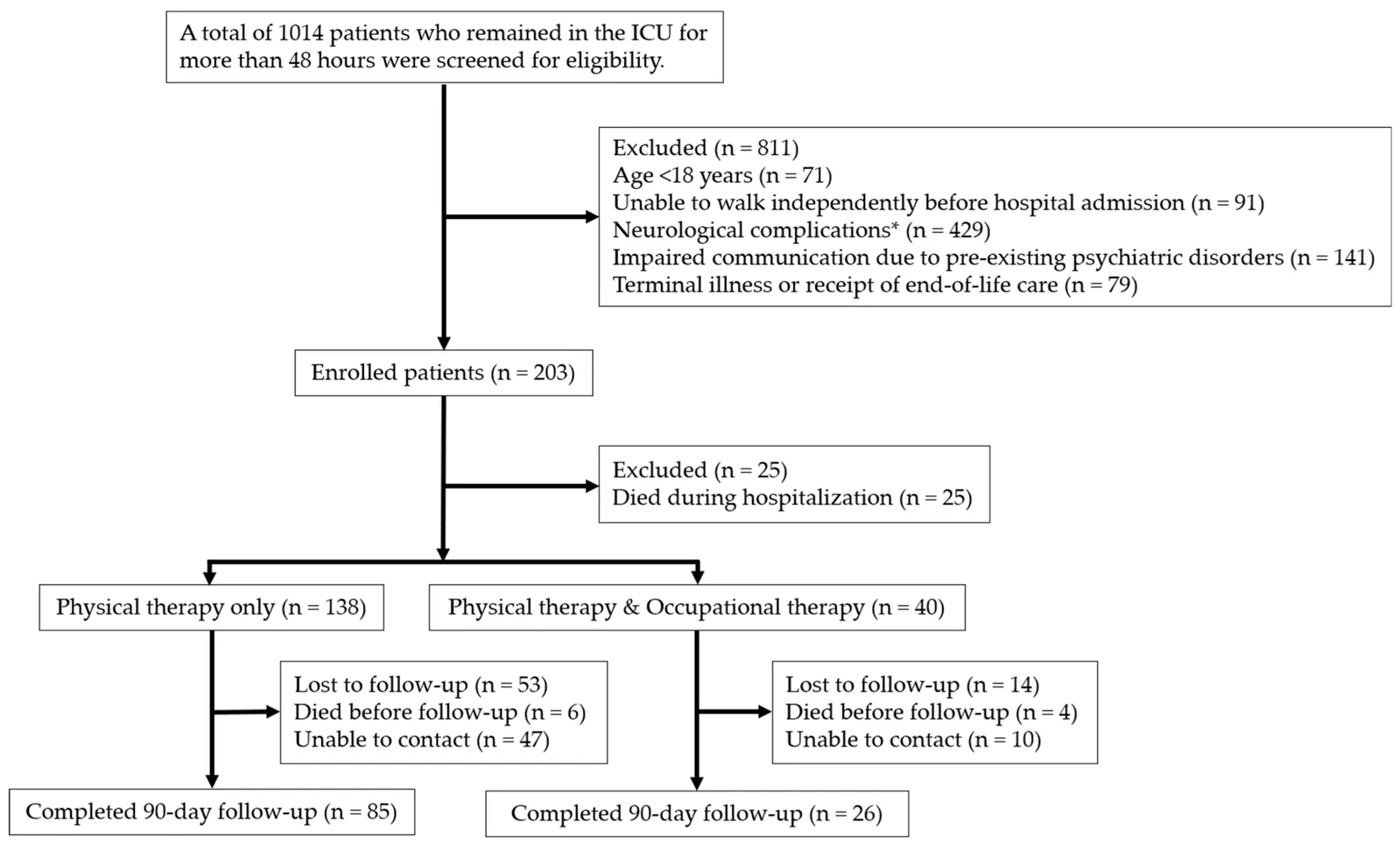
Flow diagram of patient selection and follow-up. A total of 178 patients were included in the discharge analysis, and 111 completed the 90-day follow-up analysis. * Neurological complications included cerebral infarction, cerebral hemorrhage, acute subdural hematoma, acute epidural hematoma, traumatic subarachnoid hemorrhage, and encephalitis.

Among the 1014 patients who remained in the ICU for more than 48 h, 203 met the eligibility criteria and were enrolled in the EMPICS study. After excluding 25 patients, who died during hospitalization, 178 patients were included in the discharge analysis (physical therapy group, *n* = 138; physical and occupational therapy group, *n* = 40). At 90 days after hospital discharge, follow-up data were available for 111 patients (physical therapy group, *n* = 85; physical and occupational therapy group, *n* = 26; Figure 1).

The median age of the study population was 70 years, and 62.9% of the patients were male. Baseline demographic characteristics, including age, body mass index, APACHE II score, SOFA score, Charlson Comorbidity Index, and pre-hospital Barthel Index, were generally comparable between the physical therapy and physical plus occupational therapy groups. However, patients who received occupational therapy tended to have a longer ICU stay and lower physical function at ICU discharge, including lower Barthel Index, MRC sum score, and handgrip strength. Furthermore, significant differences were observed in hospital length of stay (25 vs. 42 days, *p* < 0.01) and the total number of rehabilitation sessions (12 vs. 28 sessions, *p* < 0.01) between the physical therapy and physical and occupational therapy groups (Table 1). We additionally compared the baseline characteristics of participants with and without 90-day follow-up data (Table S1). The occupational therapy interventions provided during hospitalization included ADL training (*n* = 40, 100%), positioning (*n* = 38, 95%), upper-limb function training (*n* = 29, 73%), cognitive stimulation (*n* = 27, 68%), discharge planning (*n* = 19, 48%), family education (*n* = 17, 43%), environmental adaptation (*n* = 15, 38%), and delirium-oriented interventions (*n* = 15, 38%).

### 3.2. Clinical Outcomes

Regarding rehabilitation-related outcomes, the physical and occupational therapy group had lower Barthel Index scores (95 vs. 90, *p* = 0.02, Table 2) and Medical Research Council sum scores (60 vs. 49, *p* < 0.01) at hospital discharge, whereas HADS-D scores (4 vs. 7, *p* < 0.01) and HADS-A scores (3 vs. 5, *p* = 0.02) were higher. No significant differences were observed in the EQ-5D-5L index score or any of its dimensions. At 90 days after hospital discharge, HADS-A scores remained significantly higher in the physical and occupational therapy group (3 vs. 6, *p* = 0.02). No significant differences were observed in the EQ-5D-5L index score or any of its dimensions at this time point.

### 3.3. Multivariate Analysis of Receipt of Occupational Therapy

In multivariable analysis, occupational therapy intervention was independently associated with a higher EQ-5D-5L index score at hospital discharge (B = 0.13, 95% CI: 0.02 to 0.24, *p* = 0.02) (Table 3). At 90 days after hospital discharge, occupational therapy intervention was not significantly associated with the EQ-5D-5L index score (B = 0.05, 95% CI: −0.03 to 0.15, *p* = 0.23) (Table 3). After IPTW, residual imbalance remained for age (SMD = 0.33), Charlson Index (SMD = 0.41), and emergency admission (SMD = 0.21). Therefore, these variables were additionally included in a doubly robust outcome model. The effective sample size was 122.1 in the physical therapy group and 5.2 in the physical therapy plus occupational therapy group. In this model, adequate propensity score overlap was observed, and no trimming or weight truncation was performed. For the EQ-5D-5L dimensions at hospital discharge, significant differences were observed in mobility, self-care, and anxiety/depression. No significant associations were observed at 90 days after hospital discharge. The results of the analysis with additional adjustment for the number of physical therapy sessions are shown in Table S2.

### 3.4. Timing of Occupational Therapy Initiation

**Figure 2:**
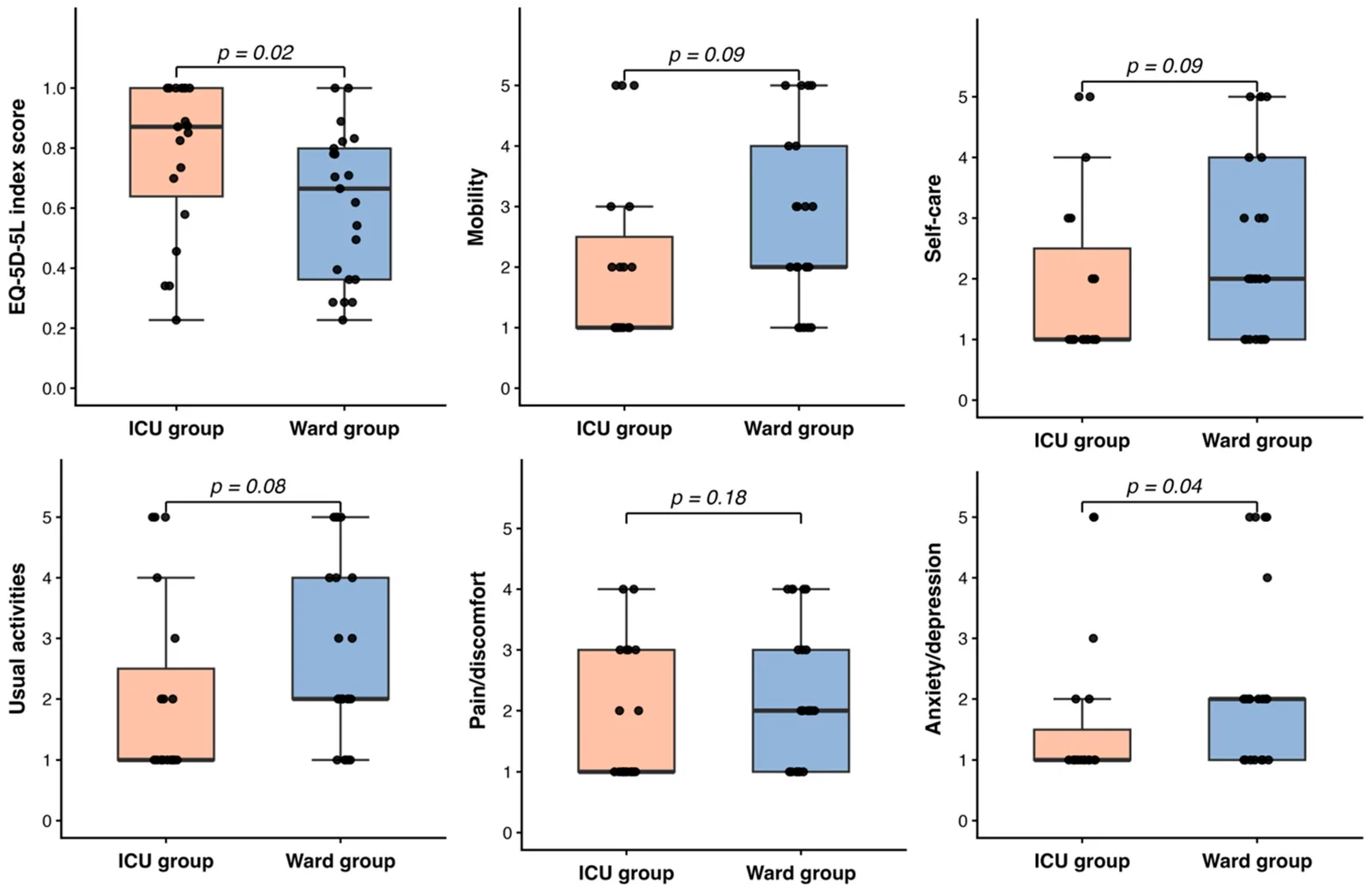
Comparison of EQ-5D-5L Index Score and Domain Between the Ward and ICU Groups. The ICU group (*n* = 19) showed a significantly higher EQ-5D-5L index score than the Ward group (*n* = 21) (0.875 [0.735–1.000] vs. 0.706 [0.507–0.816], *p* = 0.02). The ICU group also demonstrated consistently better scores across all five EQ-5D-5L domains (mobility, self-care, usual activities, pain/discomfort, and anxiety/depression), although none of the individual domain differences reached statistical significance.

Discharge outcomes were compared between patients who initiated occupational therapy during their ICU stay (ICU group, *n* = 19) and those who initiated occupational therapy after ICU discharge (Ward group, *n* = 21). The baseline characteristics were summarized in Table S3. Among patients who initiated occupational therapy in the ICU, the median time from ICU admission to initiation of occupational therapy was 2.5 days (1.8–5.3 days). The EQ-5D-5L index score was significantly higher in the ICU group than in the Ward group (0.875 [0.735–1] vs. 0.706 [0.507–0.816], *p* = 0.02). In addition, the ICU group consistently demonstrated relatively better scores across all EQ-5D-5L dimensions (mobility, self-care, usual activities, pain/discomfort, and anxiety/depression) (Figure 2). No significant differences were observed between the ICU and Ward groups in the Barthel Index (90 [70–95] vs. 85 [65–95], *p* = 0.54), HADS-A (4 [2–6] vs. 6 [4–9], *p* = 0.09), HADS-D (8 [5–10] vs. 6 [5–11], *p* = 0.30), or IES-R scores (8 [3–14] vs. 11 [6–20], *p* = 0.26).

### 3.5. Multivariate Analysis of Initiation of Occupational Therapy in the ICU

In multivariable analysis, initiation of occupational therapy in the ICU was independently associated with a higher EQ-5D-5L index score at hospital discharge (B = 0.22, 95% CI: 0.07 to 0.36, *p* < 0.01) (Table 4). At 90 days after hospital discharge, occupational therapy intervention was not significantly associated with the EQ-5D-5L index score (B = 0.05, 95% CI: −0.10 to 0.21, *p* = 0.50) (Table 4). After IPTW, residual imbalance remained for age (SMD = 0.11), APACHE II score (SMD = 0.22), Barthel Index before ICU admission (SMD = 0.34), and hospital-level differences (SMD = 0.27). Therefore, these variables were additionally included in the doubly robust outcome model. The effective sample size was 16.3 in the ICU occupational therapy group and 11.1 in the Ward occupational therapy group. In this model, adequate propensity score overlap was observed, and no trimming or weight truncation was performed. For the EQ-5D-5L dimensions at hospital discharge, significant differences were observed in mobility, self-care, usual activities, and pain/discomfort. No significant associations were observed at 90 days after hospital discharge.

## 4. Discussion

In this study, we investigated the association between acute-phase occupational therapy and QOL. Multivariable analysis demonstrated that acute-phase occupational therapy was independently associated with a more favorable EQ-5D-5L index score at hospital discharge. Occupational therapy was also associated with fewer problems in the mobility, self-care, and anxiety/depression dimensions of the EQ-5D-5L, suggesting better health status at hospital discharge. Patients who initiated occupational therapy during their ICU stay had significantly more favorable EQ-5D-5L index score at hospital discharge than those who initiated occupational therapy after ICU discharge. These findings suggest that acute-phase occupational therapy, particularly when initiated during ICU stay, may contribute to improved quality of life at hospital discharge, especially in terms of patients’ ability to perform usual daily activities.

Acute-phase occupational therapy was associated with better EQ-5D-5L at hospital discharge. This finding may be explained by the unique role of occupational therapy in addressing not only physical function but also activities of daily living, cognition, and psychological adaptation during acute hospitalization. Previous studies have shown that early physical and occupational therapy in mechanically ventilated ICU patients improves functional independence at hospital discharge and reduces delirium duration [5]. In addition, occupational therapists have specific expertise in supporting meaningful activities, ADL training, cognitive stimulation, environmental adjustment, and discharge preparation, which may directly influence patient-perceived health status and QOL [20]. Therefore, acute-phase occupational therapy may have contributed to better short-term recovery by promoting early engagement in daily activities and reducing functional and cognitive decline during hospitalization.

In contrast, acute-phase occupational therapy was not significantly associated with the EQ-5D-5L index at 90 days after hospital discharge. Although occupational therapy was associated with better QOL at hospital discharge, this short-term benefit may have diminished over time because recovery after discharge is influenced by multiple factors beyond acute hospital care, including post-discharge rehabilitation, social support, underlying comorbidities, and the natural recovery process [21]. Furthermore, the relatively small sample size and loss to follow-up at 90 days may have limited the statistical power to detect a significant association. These findings suggest that while acute-phase occupational therapy may contribute to improving QOL during hospitalization, additional interventions after discharge may be required to sustain these benefits over the longer term.

In the further analysis, occupational therapy initiated during the ICU stay was associated with more favorable QOL at hospital discharge than occupational therapy initiated after ICU discharge. This finding suggests that earlier initiation of occupational therapy may provide greater benefits for patient-reported outcomes than delayed intervention. Initiating occupational therapy during the ICU stay may facilitate earlier engagement in activities of daily living, cognitive stimulation, and participation in meaningful activities, thereby promoting functional recovery before hospital discharge. Although this further analysis was exploratory and the sample size was limited, the findings suggest a potential benefit of initiating occupational therapy during critical illness. However, given the small sample size, unadjusted comparison, and potential for confounding, these findings should be interpreted with caution. Future prospective studies are warranted to determine the optimal timing and intensity of occupational therapy and to confirm whether earlier intervention results in sustained improvements in QOL.

This study has several limitations. First, the number of patients who received occupational therapy was relatively small. In particular, the sample size at the 90-day follow-up was limited; therefore, the findings at 90 days should be interpreted with caution. Second, the specific content and intensity of occupational therapy interventions were not investigated. Third, baseline patient characteristics and disease severity differed between the groups. Although multivariable analysis was performed, residual confounding due to differences in patient characteristics may have influenced the results. Therefore, prospective randomized controlled trials are needed to confirm the causal effects of acute-phase occupational therapy. Fourth, because this was a multicenter study involving nine institutions, the criteria for initiating acute-phase occupational therapy may have varied across participating centers. Fifth, the EQ-5D-5L at hospital discharge was administered by the treating therapist, who was aware of the rehabilitation received, which may have introduced interviewer and expectation bias. Sixth, although IPTW and a doubly robust approach were used to reduce confounding, residual imbalance remained for several covariates, and the effective sample size in the physical therapy plus occupational therapy group was substantially reduced after weighting. Therefore, the results should be interpreted with caution.

## 5. Conclusions

Acute-phase occupational therapy was independently associated with better QOL at hospital discharge, particularly in the mobility, self-care, and anxiety/depression domain of the EQ-5D-5L. In contrast, no significant association was observed between occupational therapy and QOL at 90 days after hospital discharge. These findings suggest that acute-phase occupational therapy may contribute to short-term improvements in QOL among acute care patients. However, its effects on long-term QOL remain unclear, and further prospective studies are warranted to determine whether these short-term benefits can be sustained after hospital discharge.

## Figures and Tables

**Table 1:** Baseline characteristics of the study population.

Characteristics	Overall (*n* = 178)	Physical Therapy (*n* = 138)	Physical & Occupational Therapy (*n* = 40)	*p*-Value
Age, years	70 (59–78)	70 (58–78)	73 (60–82)	0.60
Sex, Male, *n* (%)	112 (62.9)	92 (66.7)	20 (50.0)	0.08
Body mass index, kg/m^2^	22.7 (20.0–25.7)	22.9 (20.3–25.8)	22.0 (18.9–24.4)	0.13
APACHE II score	19 (14–24)	19 (13–26)	19 (16–22)	0.92
SOFA score	7 (4–9)	7 (4–10)	5 (4–8)	0.30
Charlson Index	2 (1–3)	2 (1–3)	2 (1–2)	0.15
Type of ICU admission, *n* (%)				
Emergency	109 (61.2)	85 (62.0)	24 (60.0)	0.85
Scheduled	69 (38.8)	53 (38.0)	16 (40.0)	0.85
Primary diagnosis at ICU admission, *n* (%)				
Cardiovascular	78 (44.8)	69 (50.0)	9 (22.5)	<0.01
Gastrointestinal disease	47 (26.4)	33 (23.9)	14 (35.0)	0.60
Respiratory disease	24 (13.5)	20 (14.5)	4 (10.0)	0.60
Kidney disease	3 (1.7)	2 (1.4)	1 (2.5)	0.50
Cardiac arrest	5 (2.8)	3 (2.2)	2 (5.0)	0.30
Other	21 (11.8)	11 (8.0)	10 (25.0)	<0.01
ICU length of stay, days	4.8 (3.4–7.8)	4.0 (3.0–6.0)	8.5 (4.7–14.5)	<0.01
Hospital length of stay, days	27.0 (19.0–46.9)	25.0 (17.2–43.0)	42.0 (22.7–68.5)	<0.01
Pre-hospital Barthel Index	100 (100–100)	100 (100–100)	100 (100–100)	0.17
Rehabilitation sessions				
Total	14 (8–25)	12 (7–20)	28 (18–52)	<0.01
Physical therapy	12 (7–21)	12 (7–20)	15 (9–29)	0.07
Occupational therapy			13 (8–25)	

Data are presented as median (interquartile range). APACHE II, Acute Physiology and Chronic Health Evaluation II; SOFA, Sequential Organ Failure Assessment; ICU, intensive care unit.

**Table 2:** Clinical outcomes at hospital discharge and 90-day follow-up.

Outcomes	Overall (*n* = 178)	Physical Therapy (*n* = 138)	Physical & Occupational Therapy (*n* = 40)	*p*-Value
Hospital discharge				
Barthel Index	95.0 (77.5–100.0)	95.0 (80.0–100.0)	90.0 (65.0–100.0)	0.02
MRC sum score	60 (50–60)	60 (56–60)	49 (45–58)	<0.01
Handgrip strength, kg	21.0 (15.0–30.0)	21.1 (15.0–31.0)	19.6 (12.2–24.0)	0.16
HADS-A	3 (1–7)	3 (1–7)	5 (3–7)	0.02
HADS-D	5 (2–9)	4 (2–8)	7 (5–11)	<0.01
IES-R	7 (3–15)	6 (3–15)	9 (3–16)	0.60
EQ-5D-5L Index Score	0.779 (0.488–0.894)	0.772 (0.431–0.892)	0.799 (0.579–1.000)	0.31
Mobility	2 (1–3)	2 (1–4)	2 (1–3)	0.70
Self-care	1 (1–3)	1 (1–3)	1 (1–3)	0.58
Usual activities	2 (1–4)	2 (1–4)	2 (1–3)	0.17
Pain/Discomfort	2 (1–3)	2 (1–3)	1 (1–3)	0.56
Anxiety/Depression	1 (1–2)	1 (1–2)	1 (1–2)	0.10
90 days after hospital discharge				
HADS-A	4 (2–6)	3 (1–6)	6 (3–8)	0.02
HADS-D	4 (2–8)	4 (2–7)	5 (3–9)	0.10
IES-R	6 (2–13)	6 (2–12)	8 (3–14)	0.35
EQ-5D-5L Index Score	0.867 (0.703–1.000)	0.889 (0.668–1.000)	0.790 (0.703–1.000)	0.37
Mobility	1 (1–2)	1 (1–2)	1 (1–2)	0.71
Self-care	1 (1–2)	1 (1–2)	1 (1–2)	0.49
Usual activities	1 (1–2)	1 (1–2)	1 (1–2)	0.89
Pain/Discomfort	1 (1–2)	1 (1–2)	1 (1–2)	0.64
Anxiety/Depression	1 (1–2)	1 (1–2)	1 (1–2)	0.80

Data are presented as median (interquartile range). MRC sum score, Medical Research Council sum score; HADS-A, Hospital Anxiety and Depression Scale—Anxiety; HADS-D, Hospital Anxiety and Depression Scale—Depression; IES-R, Impact of Event Scale—Revised; EQ-5D-5L, EuroQol 5-Dimensions 5-Levels.

**Table 3:** Multiple regression analysis between receipt of occupational therapy and EQ-5D-5L.

Variables	Coefficient (B) or Odds Ratios (OR)	*p*-Value	95% Confidence Interval	
			Lower Limit	Upper Limit
Hospital discharge				
EQ-5D-5L Index Score	0.13	0.02	0.02	0.24
Mobility	0.31	0.01	0.12	0.78
Self-Care	0.31	0.01	0.12	0.80
Usual Activities	0.47	0.05	0.22	1.00
Pain/Discomfort	0.48	0.07	0.21	1.08
Anxiety/Depression	0.38	0.02	0.16	0.89
90 days after hospital discharge				
EQ-5D-5L Index Score	0.05	0.23	−0.03	0.15
Mobility	0.54	0.20	0.20	1.40
Self-Care	0.80	0.66	0.30	2.12
Usual Activities	0.60	0.27	0.25	1.47
Pain/Discomfort	0.53	0.17	0.21	1.31
Anxiety/Depression	0.69	0.46	0.25	1.85

Inverse probability of treatment weighting (IPTW) followed by weighted regression analysis was performed to adjust for potential confounding. Multiple linear and ordinal logistic regression analyses were used for the EQ-5D-5L index score and individual dimensions, respectively. EQ-5D-5L, EuroQol 5-Dimensions 5-Levels.

**Table 4:** Multiple regression analysis between initiation of occupational therapy in the ICU and EQ-5D-5L outcomes.

Variables	Coefficient (B) or Odds Ratios (OR)	*p*-Value	95% Confidence Interval	
			Lower Limit	Upper Limit
Hospital discharge				
EQ-5D-5L Index Score	0.22	<0.01	0.07	0.36
Mobility	0.07	0.02	0.008	0.70
Self-Care	0.06	<0.01	0.01	0.41
Usual Activities	0.09	0.01	0.01	0.62
Pain/Discomfort	0.11	0.03	0.01	0.83
Anxiety/Depression	0.07	0.08	0.004	1.45
90 days after hospital discharge				
EQ-5D-5L Index Score	0.05	0.50	−0.10	0.21
Mobility	0.55	0.56	0.07	4.02
Self-Care	0.62	0.65	0.07	4.91
Usual Activities	1.38	0.72	0.22	8.49
Pain/Discomfort	0.28	0.19	0.04	1.95
Anxiety/Depression	0.64	0.68	0.07	5.50

Inverse probability of treatment weighting (IPTW) followed by weighted regression analysis was performed to adjust for potential confounding. Multiple linear and ordinal logistic regression analyses were used for the EQ-5D-5L index score and individual dimensions, respectively. EQ-5D-5L, EuroQol 5-Dimensions 5-Levels.

## Data Availability

The data presented in this study are available from the corresponding author upon reasonable request.

## Supplementary Materials

The following supporting information can be downloaded at: https://www.mdpi.com/article/10.3390/jcm15187229/s1, Table S1: Comparison of baseline characteristics between participants with and without 90-day follow-up data. Table S2. Sensitivity analysis of the association between occupational therapy and EQ-5D-5L outcomes after additional adjustment for the number of physical therapy sessions. Table S3: Baseline characteristics of patients who initiated occupational therapy in the ICU versus after ICU discharge.

## Abbreviations

The following abbreviations are used in this manuscript:

ADL	Activities of Daily Living
APACHE II	Acute Physiology and Chronic Health Evaluation II
EQ-5D-5L	EuroQol 5-Dimensions 5-Levels
FIM	Functional Independence Measure
HADS-A	Hospital Anxiety and Depression Scale—Anxiety
HADS-D	Hospital Anxiety and Depression Scale—Depression
ICU	Intensive Care Unit
IES-R	Impact of Event Scale—Revised
MRC	Medical Research Council
PICS	Post-Intensive Care Syndrome
PTSD	Post-Traumatic Stress Disorder
QOL	Quality of Life
SF-36	36-Item Short Form Health Survey
SOFA	Sequential Organ Failure Assessment
