# Supplementary material for: Association Between Acute-Phase Occupational Therapy and Quality of Life at Hospital Discharge and 90 Days After Discharge
Source: J Clin Med. 2026 Sep 17;15(18):7229. doi: 10.3390/jcm15187229 (PMC13607415; doi:10.3390/jcm15187229)
Supplement: Supplementary file 1 [file jcm-15-07229-s001.zip › jcm-4433313-supplementary.pdf]

Supplemental material

Table S1: Comparison of baseline characteristics between participants with and without 90-day follow-up data.

Characteristics	No 90day follow up (n = 67)	Completed 90day follow up (n = 111)	*p*-value
Age, years	71 (65–78)	70 (57–78)	0.39
Sex, Male, n (%)	42 (62.7)	70 (63.6)	0.99
Body-mass index, kg/m²	22.8 (20.2–24.9)	22.7 (20–25.9)	0.69
APACHE Ⅱ score	20 (15–25)	18 (14–24)	0.28
SOFA score	7 (4–9)	7 (3–10)	0.43
Charlson Index	2 (0–3)	2 (1–2)	0.76
Type of ICU admission, n (%)			
Emergency	44 (65.7)	65 (58.6)	0.42
Scheduled	23 (34.3)	46 (41.4)	0.42
Primary diagnosis at ICU admission, n (%)			
Cardiovascular	24 (35.8)	54 (48.6)	0.11
Gastrointestinal disease	20 (29.9)	27 (24.3)	0.48
Respiratory disease	15 (22.4)	9 (8.1)	0.01
Kidney disease	1 (1.5)	2 (1.8)	0.99
Cardiac arrest	1 (1.5)	4 (3.6)	0.65
Other	6 (9.0)	15 (13.5)	0.47
Charlson Index	2 (0–3)	2 (1–2)	0.76
ICU length of stay, days	4.9 (3.3–9.0)	4.7 (3.5–7.5)	0.73
Barthel Index score at ICU discharge	25 (10–55)	40 (15–60)	0.01
Pre-hospital Barthel Index	90 (70–95)	100 (80–100)	< 0.01

Table S2. Sensitivity analysis of the association between occupational therapy and EQ-5D-5L outcomes after additional adjustment for the number of physical therapy sessions.

Variables	Coefficient (B) or Odds ratios (OR)	*p*-value	95% Confidence Interval	
			Lower limit	Upper limit
**Receipt of occupational therapy**				
Hospital discharge				
EQ-5D-5L Index Score	0.13	0.02	0.02	0.24
Mobility	0.25	< 0.01	0.10	0.66
Self-Care	0.27	< 0.01	0.10	0.71
Usual Activities	0.42	0.02	0.19	0.91
Pain/Discomfort	0.34	0.01	0.14	0.79
Anxiety/Depression	0.25	< 0.01	0.10	0.63
90 days after hospital discharge				
EQ-5D-5L Index Score	0.08	0.09	-0.01	0.18
Mobility	0.54	0.20	0.20	1.40
Self-Care	0.80	0.66	0.30	2.12
Usual Activities	0.60	0.27	0.25	1.47
Pain/Discomfort	0.53	0.17	0.21	1.31
Anxiety/Depression	0.69	0.46	0.25	1.85
**Initiation of occupational therapy in the ICU**				
Hospital discharge				
EQ-5D-5L Index Score	0.17	0.01	0.02	0.33
Mobility	0.11	0.07	0.01	1.27
Self-Care	0.11	0.02	0.01	0.75
Usual Activities	0.26	0.19	0.03	1.96
Pain/Discomfort	0.29	0.29	0.02	2.93
Anxiety/Depression	0.15	0.23	0.006	3.39
90 days after hospital discharge				
EQ-5D-5L Index Score	0.04	0.62	−0.12	0.21
Mobility	0.68	0.71	0.09	5.23
Self-Care	0.75	0.80	0.08	7.04
Usual Activities	1.42	0.72	0.20	10.12
Pain/Discomfort	0.30	0.25	0.04	2.31
Anxiety/Depression	0.66	0.72	0.06	6.72

Table S3: Baseline characteristics of patients who initiated occupational therapy in the ICU versus after ICU discharge

**Characteristics**	**Overall (n = 40)**	**ICU group (n = 19)**	**Ward group (n = 21)**	***p*-value**
Age, years	73 (60–81)	69 (57–81)	76 (61–82)	0.29
Sex, Male, n (%)	21 (52.5)	9 (47.4)	12 (57.1)	0.52
Body-mass index, kg/m^2	22.0 (18.9–24.4)	21.2 (18.8–23.8)	22.9 (19.1–24.4)	0.33
APACHE II score	19 (16–22)	18 (16–21)	20 (17–23)	0.27
SOFA score	5 (4–8)	5 (3–8)	7 (5–9)	0.09
Charlson Index	1 (0–2)	1 (0–2)	2 (1–2)	0.99
Type of ICU admission, n (%)				
Emergency	24 (60)	12 (63.2)	12 (57.1)	0.75
Scheduled	16 (40)	7 (36.8)	9 (42.9)	0.75
Primary diagnosis at ICU admission, n (%)				
Cardiovascular	9 (22.5)	3 (15.8)	6 (28.6)	0.45
Gastrointestinal disease	14 (35)	8 (42.1)	6 (28.6)	0.51
Respiratory disease	4 (10)	2 (10.5)	2 (9.5)	0.99
Kidney disease	1 (2.5)	1 (5.3)	0 (0)	0.47
Cardiac arrest	2 (5)	2 (10.5)	0 (0)	0.21
Other	10 (25)	3 (15.8)	7 (33.3)	0.27
Charlson Index	1 (0–2)	1 (0–2)	2 (1–2)	0.99
ICU length of stay, days	8 (4.7–14.5)	8 (4.5–15)	9 (5–14)	0.62
Length of stay in hospital, days	42 (22.7-68.5)	30.8 (22-58.9)	44 (28-86)	0.23
Barthel Index score at ICU discharge	30.0 (12.5–42.5)	30 (20–55)	25 (5–40)	0.15
Pre-hospital Barthel Index	100 (100–100)	100 (80–100)	100 (100–100)	0.14
